# Large-Area Resistive Strain Sensing Sheet for Structural Health Monitoring

**DOI:** 10.3390/s20051386

**Published:** 2020-03-03

**Authors:** Levent E. Aygun, Vivek Kumar, Campbell Weaver, Matthew Gerber, Sigurd Wagner, Naveen Verma, Branko Glisic, James C. Sturm

**Affiliations:** 1 Department of Electrical Engineering, Princeton University, Princeton, NJ 08544, USA; campbellweaver@mac.com (C.W.); wagner@Princeton.EDU (S.W.); nverma@princeton.edu (N.V.); sturm@princeton.edu (J.C.S.); 2 Department of Civil and Environmental Engineering, Princeton University, Princeton, NJ 08544, USA; vivekk@princeton.edu (V.K.); gerberbeam@gmail.com (M.G.); bglisic@Princeton.EDU (B.G.)

**Keywords:** structural health monitoring, strain sensing sheet, large area electronics, resistive strain gauges

## Abstract

Damage significantly influences response of a strain sensor only if it occurs in the proximity of the sensor. Thus, two-dimensional (2D) sensing sheets covering large areas offer reliable early-stage damage detection for structural health monitoring (SHM) applications. This paper presents a scalable sensing sheet design consisting of a dense array of thin-film resistive strain sensors. The sensing sheet is fabricated using flexible printed circuit board (Flex-PCB) manufacturing process which enables low-cost and high-volume sensors that can cover large areas. The lab tests on an aluminum beam showed the sheet has a gauge factor of 2.1 and has a low drift of 1.5 μϵ/day. The field test on a pedestrian bridge showed the sheet is sensitive enough to track strain induced by the bridge’s temperature variations. The strain measured by the sheet had a root-mean-square (RMS) error of 7 $\mu \epsilon_{rms}$ compared to a reference strain on the surface, extrapolated from fiber-optic sensors embedded within the bridge structure. The field tests on an existing crack showed that the sensing sheet can track the early-stage damage growth, where it sensed 600 μϵ peak strain, whereas the nearby sensors on a damage-free surface did not observe significant strain change.

## 1. Introduction

The aging infrastructure of the United States is in dire need of maintenance and repairs. The ASCE 2017 report-card evaluates the current infrastructure health as D+. The expected cost of repair and maintenance of the existing infrastructure is around $5 trillion [1]. Appropriately, structural health monitoring (SHM) systems are increasingly being incorporated in new projects and added to existing structures with goals to identify the damage initiation and propagation [2] or analyze the strength reserves [3]. Timely information from a monitoring system helps in condition-based maintenance, reducing the life-cycle cost of the structure [4,5,6].

The degradation of civil engineering structures often presents itself in the form of cracks, excessive deformation, or spalling, and are a result of stresses exceeding the strength or serviceability limits. Local properties such as strain and wave velocities or global properties such as vibration modes, display a measurable change in response to the degradation and are commonly used as damage indicators [7]. Strain-based monitoring is widely used in the structural health monitoring community and is the basis of this work [8]. The underlying principle behind strain-based monitoring is that any significant change in a structure’s behavior results in a measurable perturbation in the strain field. In practice, to capture these changes, monitoring systems rely on the damage either physically affecting the sensor (for example, a crack crossing over) or being close enough such that the strain change cannot be attributed to environmental or loading conditions. Placing sensors in direct contact with damage offers high sensitivity. Currently available sensors used for direct sensing could be considered as point sensors or one-dimensional sensors. These include short-gauge, long-gauge, or distributed sensors in the increasing order of spatial coverage. As an example, the fiber optic sensors are typically placed on a straight line (i.e., one dimensional) throughout the structure and enable sensing over very large distances [9,10,11,12]. However, they also have a high spatial selectivity and generate a large signal only when the damage is nearby. A similar issue in sensitivity to damage applies to other types of commercially available strain sensors. Thus, a reliable damage detection requires sensors to be in the close proximity of the damage [13], which can be achieved via a “sensing sheet” design with a two-dimensional (2D) sensor array that covers a large area with a dense network of sensors. Additional benefits of having such a dense coverage of the structure are (1) the sensors don’t need to measure strains very accurately as long as they capture the anomalous behavior reliably and, (2) the overall system is more robust and resilient to a few malfunctioning sensors.

Given the advantages of two-dimensional sensing systems, various research groups have developed prototypes for such sensors in the past two decades. A nonexhaustive list includes the following: (1) Bioinspired carbon-nanotube-based sensing system, developed by [14] for strain monitoring and impact damage identification. These sensing skins are tailored for specific electromechanical and electrochemical mechanisms for strain and corrosion monitoring [15,16]. Laboratory tests showcased the strain sensing and damage detection capabilities, and these sensors show promise for long term structural health monitoring applications. Electrical impedence tomographical (EIT) conductivity mapping allowed for identification of damage location and quantification of damage severity. EIT-based sensing has demonstrated damage detection capability for concrete structures [17]. (2) Piezoelectric (PZT) sensing skins comprising of transducers and receivers built into a flexible layer have been proposed for damage detection for aeronautical applications [18,19] as they provide wireless network capabilities [20]. Numerical simulations and laboratory experiments have validated the use of such sensors for wind turbine blade monitoring [21]. (3) A robust and durable sensing skin based on large-area capacitor—called a soft elastomeric capacitor (SEC)—that can measure additive strain on the structure and have been characterized for static as well as dynamic sensing capabilities through laboratory experiments [22,23,24,25,26,27]. Through further numerical and laboratory experiments, the SEC-sensing skin have been demonstrated to perform damage detection and localization on a wind turbine blade. (4) Recently, graphite-based strain sensor arrays have been demonstrated for SHM application [28,29,30]. To compensate for the temperature sensitivity of graphite, a full Wheatstone bridge design was implemented and laboratory tests validated the dynamic measuring capabilities of the sensor array. The sensing system was tested on a highway bridge to measure the dynamic response of the traffic and measurements carried out for a 60-s duration. Other notable works have used photonic crystals [31], carbon-nanotube composite crystals [32], and microfabricated sensor networks [33] to achieve two-dimensional sensing networks. In previous works performed at Princeton University, we developed a thin-film two-dimensional sensing sheet as one possible two-dimensional sensing system using an array of resistive strain gauges, where commercial resistive sensors were wired one by one to a flexible sheet with metal interconnect traces [34]. It was also integrated with scanning, readout, and power management subsystems implemented by a combination of flexible thin-film circuits and efficient CMOS circuits. Although the manual integration of sensors was sufficient for proof-of-concept laboratory experiments, it was not a scalable approach for real-life applications.

All above presented research is still ongoing and has great promise for future applications. The main challenge it faces is scalability to the size of civil structures. This study tries to address this gap by presenting a scalable approach, a large-area resistive strain sensing sheet design fabricated using the flexible printed circuit board (Flex-PCB) manufacturing process. The Flex-PCB industry offers a sensor manufacturing process that is already optimized for low-cost and high-volume production of large (few $m^{2}$) sensing sheets [35]. Section 2 describes the operation and design of the sheet. It is integrated with CMOS-based biasing, readout, control, and computation subsystems. The details and design choices of these subsystems are explained in Section 3. The sheet’s laboratory characterization and the field tests are discussed in Section 4.

## 2. Strain Sensing Sheet Technology

The strain sensing sheet is a 2D thin-film resistive strain sensor array with 8 elements fabricated on a flexible substrate. The sheets were manufactured by a commercial PCB manufacturer, Q-Flex Inc., using a standard manufacturing process. While the designed and purchased sheets cover 7.8 cm × 19.6 cm, the vendor can supply sheets up to 30.5 cm × 292 cm.

### 2.1. Sensing with Thin-Film Resistive Strain Gauges

**Figure 1:**
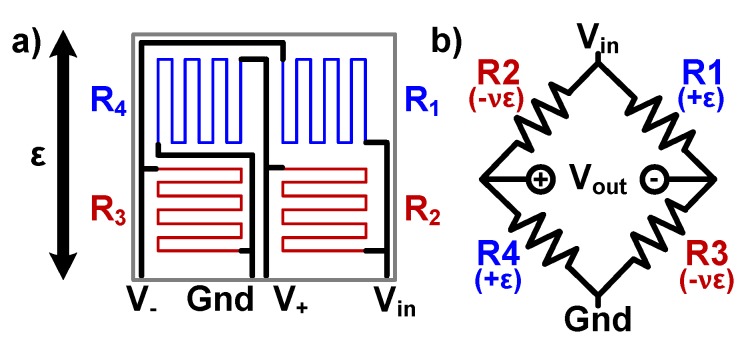
A resistive strain sensing gauge under axial strain. (**a**) The sensor layout where piezoresistive trace directions and the direction of strain are depicted, with blue predominately parallel to the strain and red perpendicular to it. (**b**) The corresponding circuit diagram of the sensor, a full Wheatstone bridge structure. In parenthesis, mechanical strain on each resistor is shown.

Electrical resistance of metals change under mechanical strain, and this effect can be utilized to measure the strain by laminating a flexible sheet (e.g., polyimide) with metal traces to the surface to be measured. Resistive strain gauges typically have serpentine-shaped long metal traces, patterned as shown in Figure 1. The sensitivity to strain is described with the term gauge factor (GF) and is given by

(1) $$GF=\frac{\Delta R/R}{\Delta L/L}=\frac{\Delta R/R}{\epsilon }=1+2\nu_{m}+\frac{\Delta \rho /\rho }{\epsilon },$$

where *R* and *L* are the initial electrical resistance and the length of a metal trace, ΔR and ΔL represent the change in these terms, $\nu_{m}$ is the Poisson’s ratio of the metal, and Δρ/ρ is the resistivity change due to the piezoresistive effect for a mechanical strain (ϵ) parallel to the metal traces.

The full Wheatstone bridge structure, shown in Figure 1, is used to translate the strain-induced resistance change to a voltage difference. The output to input voltage ratio, $V_{r}$, is defined as

(2) $$V_{r}=\frac{V_{out}}{V_{in}}=\frac{R_{4}}{R_{4}+R_{2}}-\frac{R_{3}}{R_{3}+R_{1}}.$$

The sensor was designed in such a way that initially all the resistors have the same value, yielding $V_{r}$ of 0 V. In case of a temperature change, all resistors experience the same resistance change, so $V_{r}$ stays at 0 V. This means that the sensor is virtually insensitive to temperature (i.e., it is self-compensated), but it is also insensitive to thermal strain in the structure. However, under mechanical axial strain with the direction shown in Figure 1b, the new resistance and $V_{r}$ values are given by

(3) $$\begin{array}{c} R_{1,4(parallel)}=R\left(1+\epsilon GF\right), \end{array}$$

(4) $$\begin{array}{c} R_{2,3(perpendicular)}=R\left(1-\nu \epsilon GF\right), \end{array}$$

(5) $$\begin{array}{c} V_{r}=\frac{R(1+\epsilon GF)}{R(2+(1-\nu )\epsilon GF)}-\frac{R(1-\nu \epsilon GF)}{R(2+(1-\nu )\epsilon GF)}, \end{array}$$

(6) $$\begin{array}{c} V_{r}=\frac{(1+\nu )\epsilon GF}{2+(1-\nu )\epsilon GF}, \end{array}$$

where ν is the Poisson’s ratio of applied surface. By measuring $V_{r}$, the axial strain on the sensor can be calculated using the following equation:

(7) $$\epsilon =\frac{2V_{r}}{GF[\left(1+\nu \right)-\left(1-\nu \right)V_{r}]}.$$

A crack can occur in any random orientation, and the same crack can generate different $V_{r}$ output depending on its overlap and orientation with respect to the sensor [36]. Thus, early-stage crack detection applications focus on detecting sudden and large changes (i.e., anomalies) in the strain of at least 40–100 μϵ, typically over 1000 μϵ (1000 × 10${}^{-6}$ m/m) [37].

### 2.2. Strain Sensing Sheet Fabrication and Design

**Figure 2:**
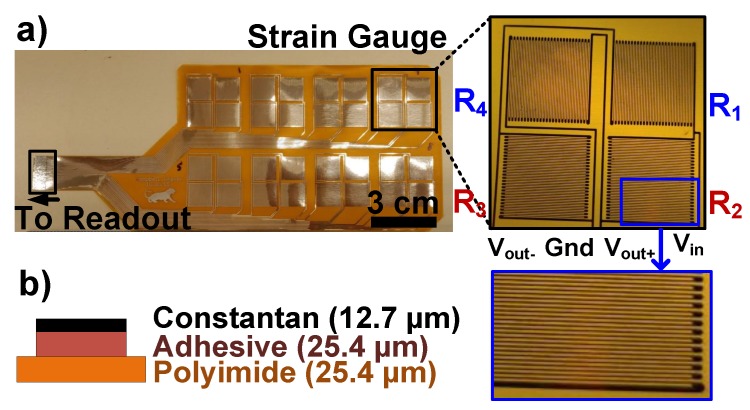
(**a**) Photographs of the thin-film strain sensing sheet. (**b**) The material stack-up of the sheet.

The sensing sheet prototype has eight strain sensing elements as shown in Figure 2. The sheet was designed at Princeton University and fabricated by a flexible PCB manufacturer, Q-FLEX Inc. The fabrication starts with a commercial foil with a material stack-up of polyimide (25.4 μm), adhesive (25.4 μm), and constantan (Cu-Ni alloy) (12.7 μm). First, the image of the desired layout pattern was transferred to the sample using a photolithography process. Then, unwanted constantan regions were removed by etching. Next, the sample was laser cut to the designed shape. Finally, a 0.3-mm-thick stiffener layer was glued to the edge which will be inserted to a zero insertion force (ZIF) connector on the rigid readout PCB.

Constantan was chosen as the strain sensing conductor because of its low temperature coefficient of resistance of ±20 ppm/°C, which further reduces a potential measurement drift due to ambient temperature fluctuations [38]. The trace width and the spacing between traces were chosen as 100 μm which were set by the minimum feature size achievable by the manufacturer’s lithography capabilities. Reducing them further would increase sensor resistance. This is preferred because the effect of the lead wires on the measurement and the power consumed by the sensors would be minimized for a fixed input bias voltage. The area of each resistor in the gauge is 10.8 mm × 10.8 mm and has 300-ohms resistance under no strain. The measured strain value represents the average strain over the area covered by the gauge; not the peak strain due to a crack. The choice of gauge length and the placement of the gauges in the array are beyond the scope of this study and need to be chosen based on the target application requirements. Previous studies on optimal sensor arrangement to increase the probability of detecting cracks can be used as a guideline [13,26]. The gauge dimensions presented here can be scaled as desired. The total strain sensing sheet size, including interconnects, is 19.6 cm × 7.8 cm.

## 3. System Overview

**Figure 3:**
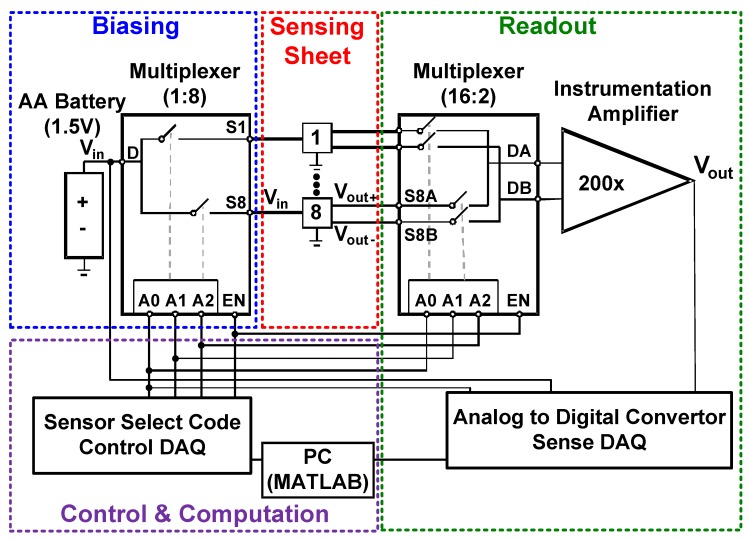
The circuit diagram of the large-area strain sensing system where biasing, strain sensing sheet, readout, and control subsystems are highlighted.

**Figure 4:**
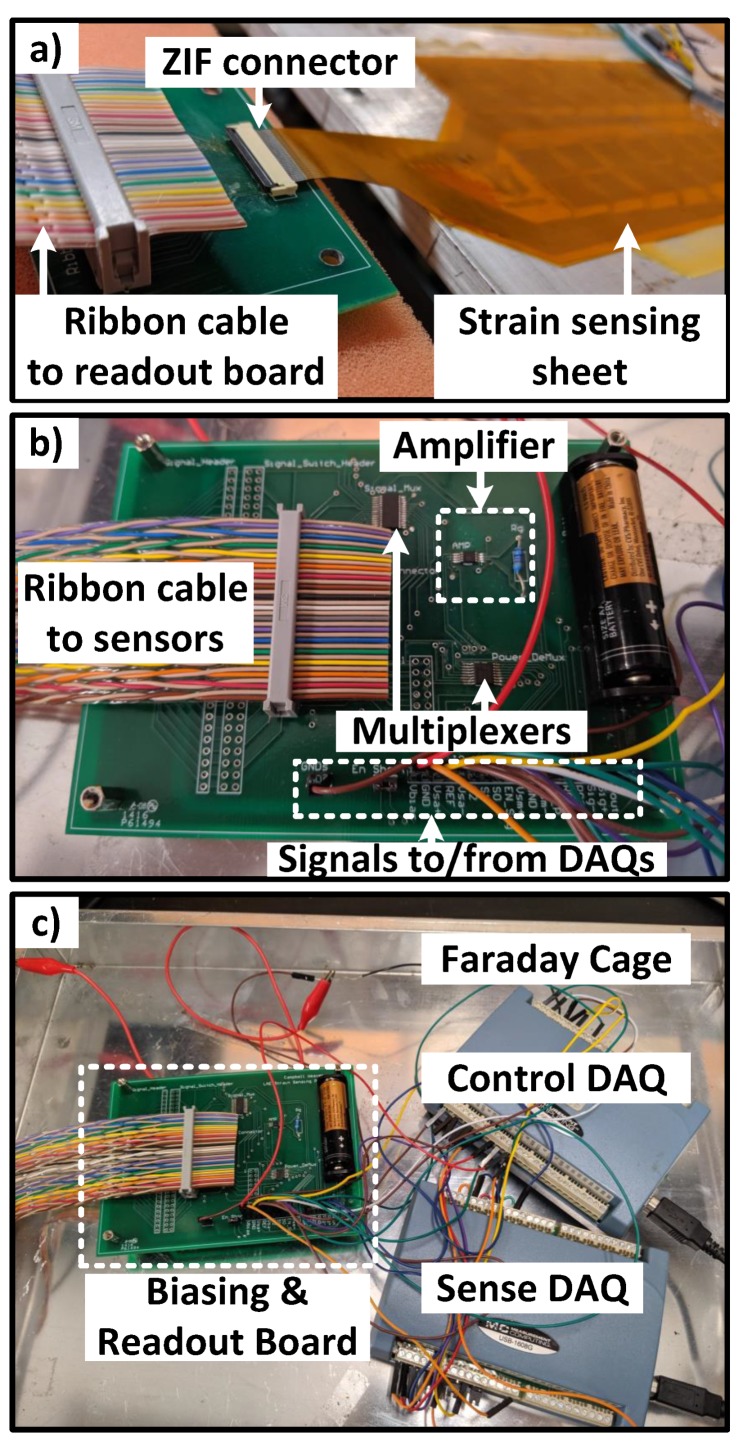
Measurement setup. (**a**) Strain sensing sheet glued to the aluminum beam. A ZIF connector was used to connect the sheet to a rigid PCB for readout. (**b**) The biasing and readout PCB with an amplifier and multiplexers are shown. A ribbon cable was used to bias and receive signals from the strain sensing sheet. (**c**) Readout and control boards were kept inside a metal Faraday cage to minimize electrical noise.

The strain sensing system consists of (1) biasing, (2) sensing sheet, (3) readout, (4) control and computation subsystems, as shown in Figure 3. To conserve power, the biasing circuitry selects and powers up a single strain sensor at a time based on a control signal. Then, the differential voltage signal from the sensor is amplified, digitized, and converted to a strain value by the readout and computation subsystems. For rapid prototyping and debugging purposes, only the sensing sheet was fabricated on a flexible substrate, rigid circuit boards and components were used for the other circuit subsystems, as shown in Figure 4. However, in the final product, these circuits can be directly fabricated on a single flexible sensing sheet using low-temperature thin-film circuits [34] or they can be flip-chip bonded on it.

### 3.1. Biasing Circuit

Resistive strain sensors can be powered up with an alternating (AC) or a constant (DC) voltage bias. Using an AC bias would mitigate the effects of Flicker (1/f) and line (60 Hz) noises due to long lead wires [39]. Although the proposed system is compatible with an AC supply, a DC voltage bias was chosen to ease the system implementation. A battery with 1.5 V initial output voltage and a capacity of 2000 mAh was used. To ease fabrication, constantan traces with high resistivity are used for the interconnects too. The voltage drop on them are considered while calculating the strain values. Under this biasing condition, the sensor resistance of 300 Ω consumes 7.5 mW power. When the sensors are continuously scanned, the battery can last up to 400 h. Scanning all sensors takes 8 s; if the sensors are scanned only once per minute, the battery life can go for up to 3000 h (125 days).

To conserve power, an analog multiplexer (Analog Devices, ADG1606, Figure 3) was used to power up only one sensor at a time. While the enable (EN) signal is high (5 V), the multiplexer connects its input (D) to one of the 8 outputs (S1-8) based on the 3-bit binary select code (A[2:0]). The power consumption can be reduced further for periodic measurements by disconnecting the battery from the sensors via setting the enable signal to low (0 V) between measurement cycles.

### 3.2. Readout Circuit

The differential output signal of the selected strain sensor, $V_{out}$, was connected to an instrumentation amplifier via a differential analog multiplexer (Analog Devices, ADG1607), as shown in Figure 3. This multiplexer chooses among eight differential signal pairs based on the 3-bit binary select code (A[2:0]) and connects the desired sensor to its output (DA, DB). $V_{out+}$ and $V_{out-}$ had a large common voltage of $V_{in}/2$ (750 mV) and a small voltage difference (few mV or less) due to the sensed strain. The differential signal was amplified with the instrumentation amplifier, Analog Devices AD8227. Its high common-mode rejection ratio of 140 dB enables operation under a large common mode signal and its high input impedance of 0.8 GΩ creates a negligible loading effect on the sensor. The amplifier has a variable differential mode gain ranging from 5 to 1000 which is set by an external resistor. A voltage gain of 200 was chosen to accommodate large strain values (up to 25 mϵ) and initial strain offsets due to the installation process.

The output of the instrumentation amplifier and the battery’s output voltage were sampled with an analog input data acquisition (DAQ) board, MCC USB-1608G (Sense DAQ in Figure 3). It has an input voltage range of ±10 V and 16-bit resolution. It uses a single analog-to-digital converter and can sample up to 16 single-ended channels. The input signals were sampled at 10 kHz, but the mean of over 1 s long measurement segments were taken to average out the line (60 Hz) noise.

### 3.3. Control Circuit and Computation

An analog control DAQ board, MCC USB-3105 (Control DAQ in Figure 3), was used to control multiplexers by generating the EN and the 3-bit binary select signals. Each sensor in the array was sensed for 1 s, so the complete scan takes 8 s. The least significant bit of binary select signal (A0) was used to synchronize analog control and sense DAQs. A MATLAB script was used to control DAQs and compute the strain value according to Equation (7).

The readout and biasing circuits are implemented on a rigid board and connected to the sensing sheet using a ZIF connector, to ease prototyping. In our experiments, this connector caused connection problems between the sensors and readout circuitry. However, this can be prevented in a final commercial product by integrating the readout circuitry on the same single large flexible sensing sheet. The control and sense DAQs can readout from 15 such sensing sheets simultaneously, which would cover 2.3 m${}^{2}$. Our manufacturer’s single sheet size limit is 30.5 cm × 292 cm, so larger single sheet designs are commercially possible.

## 4. Measurement Results

First, the gauge factor and the stability of the sensing sheet were characterized on an aluminum cantilever beam. Then, the field test was conducted, where the sheet was installed on an instrumented pedestrian bridge.

### 4.1. Installation and Measurement Methods

The installation began with mechanically abrading the target surface using sand paper and degreasing with acetone. Next, the sensor was applied to surface using a thin layer of Araldite 2012. Finally, it was fixed with a duct tape for a stable curing process for the next 24 h at ambient temperature.

The choice of adhesive has an impact on strain transfer characteristics. Araldite 2012 was preferred because of good strain transfer characteristics [40]. Air bubbles trapped between the sensor and the target surface would prevent the strain transfer. The large surface area of the sheet makes this problem even more pronounced; therefore, a few small holes were punched on regions without metal traces in order to release the trapped air.

The strain measurement procedure began with a calibration measurement, where each sensor’s initial strain value was recorded and used as a baseline value (i.e., zero-strain level). The baseline signals were not exactly zero due to small mismatches in the Wheatstone bridge resistors and the adhesive shrinkage during the curing process [41]. The strain values reported in this work were calculated relative to the baseline measurements using Equation (7).

### 4.2. Sensor Characterization

**Figure 5:**
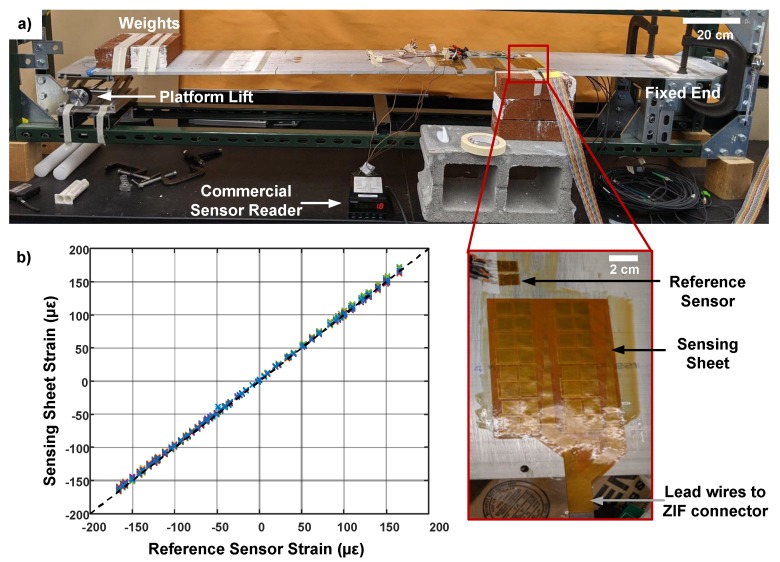
Sensing sheet gauge factor characterization. (**a**) The experiment setup, an instrumented aluminum cantilever, used to compare our sensing sheet performance with a reference thin-film strain sensor. Platform lift is used to adjust deformation of the beam. Inset shows the placement of the reference sensor and sensing sheet. (**b**) The strain measured by the sensing sheet and reference sensor. Colored x marks represent the measurements from eight sensors on the sensing sheet. The dashed curve is added to show the ideal y = x line.

The gauge factor of 2.1 is assumed for the sensing sheet based on the values reported for the strain sensitive layer, constantan [42]. To validate this assumption, the sheet’s strain outputs were compared with a commercial strain gauge, Omega SGT-4, using the cantilever test setup shown in Figure 5a. The dimensions of the aluminum beam were 170 cm × 25.4 cm × 1 cm. One end of the beam was clamped to a table and the sensors were installed 37.5-cm away from the fixed end. The other end was placed on a platform lift. Weights were added to bend the beam to achieve a wider range of tensile strain on sensors. First, a baseline measurement was taken to remove the offset signals. Then, the strain on the sheet was measured when the platform lift was adjusted to have 5–10 μϵ change in strain on the reference sensor. Figure 5b shows that the reference sensor and sensing sheet output the same strain value under the same condition. This validates our gauge factor assumption of 2.1. As mentioned before, the goal of the sensing sheet is detecting sudden and large changes in the strain field due to damage on the structure, rather than becoming a precise strain measurement tool. In practice, orientation and path of a crack will be random, therefore our uniform axial strain assumption may not be valid, but the sensing sheet would still output a significant strain change so that it can reliably detect early stage damage and call for further inspection.

**Figure 6:**
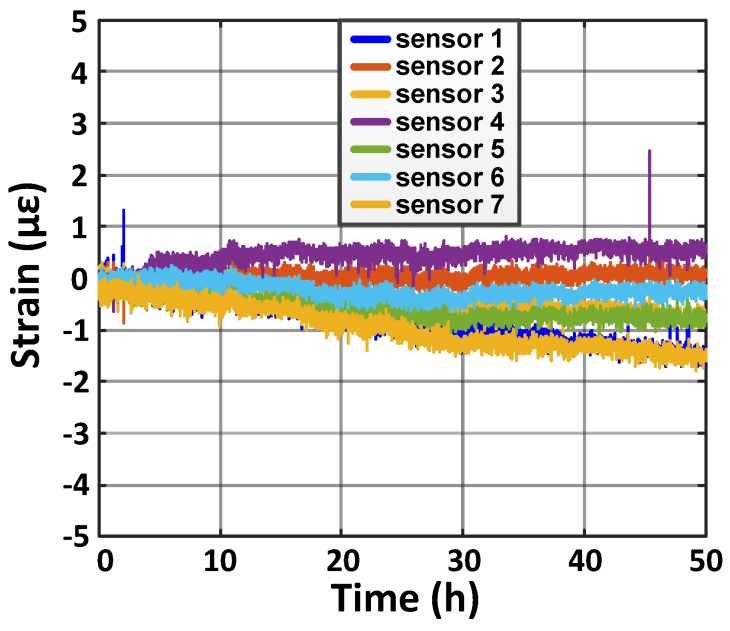
Sensor stability test of the sensing sheet shows less than 3 μϵ drift in continuously measured strain over 50 h. One sensor is omitted since it had a ZIF connector problem. Both ends of the beam were clamped.

The stability of the sensing sheet was investigated using the same beam with both ends fixed and no weight applied. Strain values drifted less than 3 μϵ over 2 days (i.e., 1.5 μϵ per day), shown in Figure 6. A typical crack would generate at least 40–100 μϵ, typically over 1000 μϵ [37], so the observed strain drift would not affect the detection of a crack formation even when the sensors are measured only periodically, e.g., once a day.

### 4.3. Field Test Without a Crack

**Figure 7:**
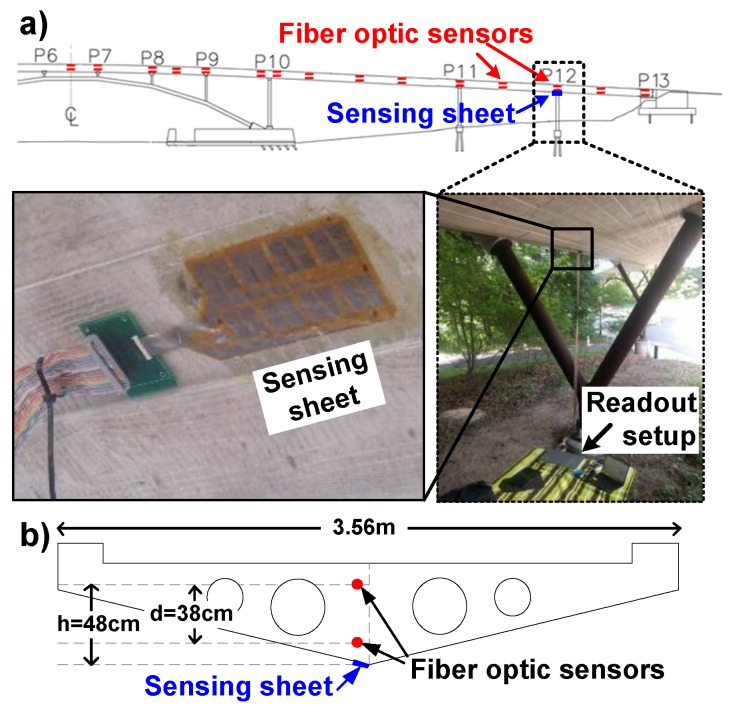
The field-test setup on Streicker Bridge. (**a**) The schematic showing locations of reference fiber-optic sensors and sensing sheets. Photographs of the installed sensing sheets and test setup below P12 location. (**b**) The cross-section of the bridge showing sensor placement.

The strain sensing sheet was tested on an instrumented pedestrian bridge, the Streicker Bridge at Princeton University campus. The aim of the test was to assess the performance of sensor in real-life settings, including the strain transfer from the structure to sensing sheet. The sheet was installed at a location depicted as P12 (Pier 12) in Figure 7a. Long-gauge fiber-optic sensors, based on Fiber Bragg gratings, were used as a reference. They were embedded into the concrete during the construction at locations shown in Figure 7b. Therefore, they do not directly measure the strain on the surface. Assuming a linear strain profile, the strain near the sheet can be calculated by

(8) $$\epsilon_{surface}=\epsilon_{top}+\frac{h\times (\epsilon_{bottom}-\epsilon_{top})}{d},$$

where $\epsilon_{bottom}$, $\epsilon_{top}$ are the strain measured by the fiber-optic sensors, and d and h are distances between sensors, as shown in Figure 7a.

**Figure 8:**
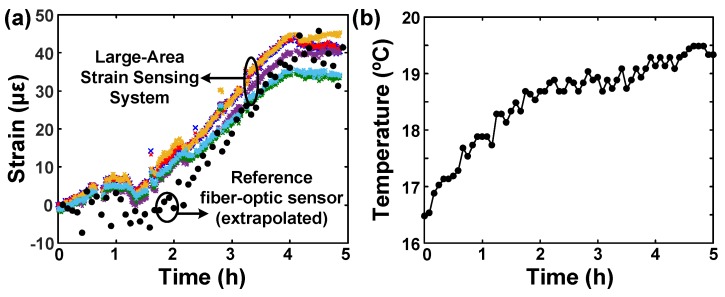
(**a**) Strain and (**b**) temperature measurement results taken from the bridge over 5 h (9 am to 5 pm). The slow tensile strain increase is because of the temperature rise from morning to afternoon. Different colors represents 6 sensors on the sensing sheet. The temperature was measured at the top fiber-optic sensor location.

The temperature variations of the bridge during the day were the main source of strain changes. The bridge is statically indeterminate, the temperature changes create both thermal strain and mechanical strain. The bridge had a low pedestrian traffic which did not generate a significant dynamic strain signal. The strain and temperature on this location were measured every 5 min by the fiber-optic sensor setup. Figure 8 shows measured mechanical strain at the the bottom surface, which was monitored continuously using the sensing sheet for 5 h (from 9 am to 2 pm). Note that the bridge heats up nonuniformly, and the temperature provided here was measured by the top fiber-optic sensor. Strain does not change simply linearly with the temperature due to the temperature gradients in the structure [43], but still generates strain that can be detected by the sensing sheet. Only 6 out of 8 sensors were recorded due to a connection problem with the ZIF connectors between the sensing sheet and the readout circuitry. The root-mean-square (RMS) error between the reference strain (extrapolated from fiber-optic sensors’ data) and the strain measured by 6 sensors on the sheet is 7 $\mu \epsilon_{rms}$. Given that the main aim of sensing sheet is not accurate strain measurement, but rather damage detection, this error is considered small and acceptable. Thus, the experiment in general confirms good strain transfer from structure to sensing sheet and good performance of the latter in real-life settings.

### 4.4. Field Test on an Existing Crack

**Figure 9:**
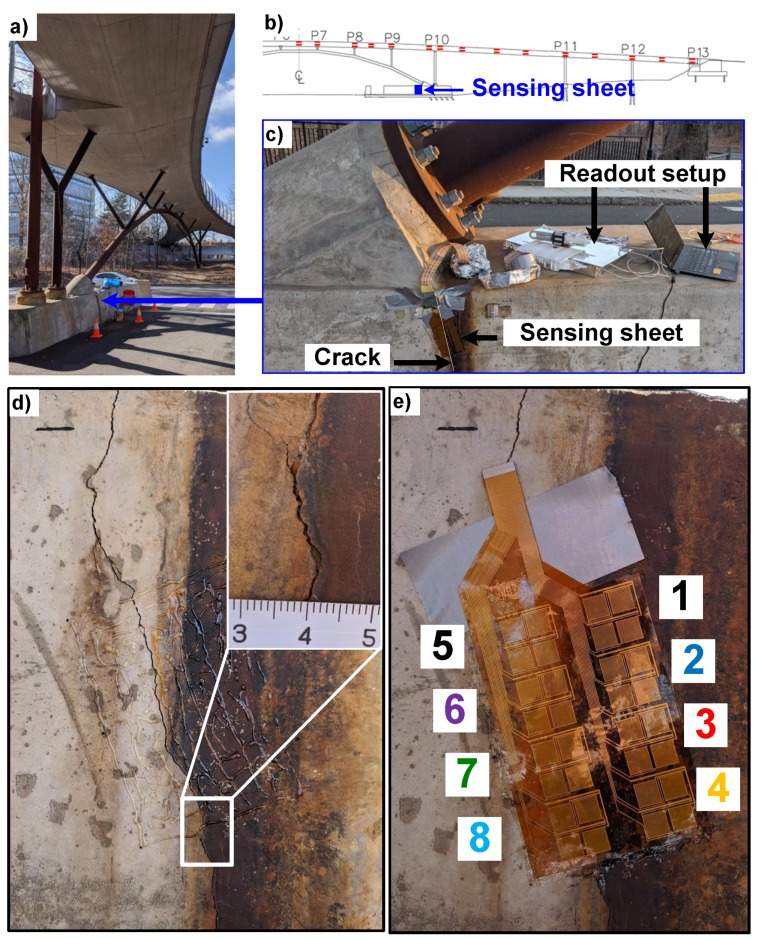
Placement of a sensing sheet on an existing crack on the foundation of pedestrian bridge. (**a**) Picture of the bridge. (**b**) Schematic showing the location of the sensing sheet. (**c**) Picture showing the location of crack with respect to the bridge support. (**d**) Picture of the crack, inset shows portion of the crack where crack size is about 0.5 mm. (**e**) Picture of the laminated sensing sheet over the crack. Only sensors 5–8 are placed on the crack.

The sensing sheet is tested for its damage detection functionality on the same bridge by monitoring the behavior of existing cracks. As mentioned previously, the dynamic load on the bridge due to pedestrians is negligible. The only existing cracks on the bridge are shrinkage cracks visible on the foundation, as shown in Figure 9a–c. The initial crack opening is about 0.5 mm and its path is shown in Figure 9d. The sensing sheet is installed so that only sensors 5-8 would be on the crack and sensors 1–4 would be the control group glued to a crack-free surface experiencing the same ambient conditions.

**Figure 10:**
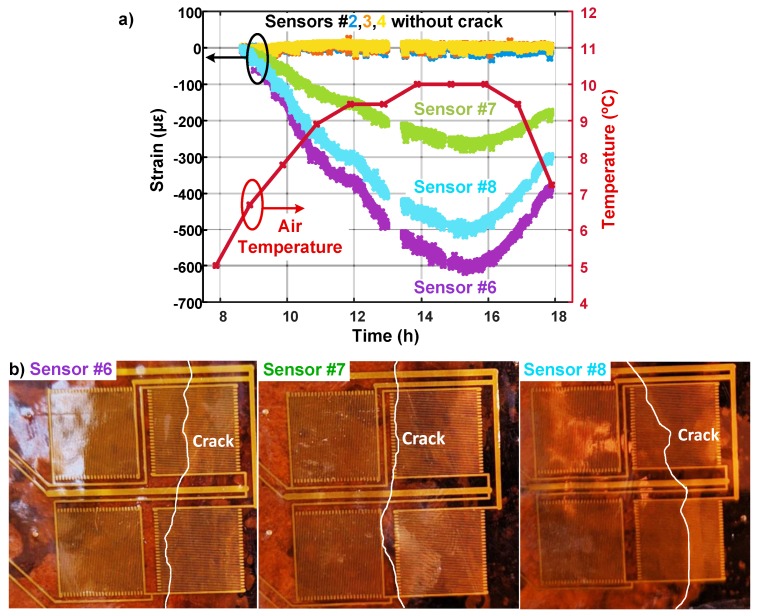
(**a**) Strain and temperature measurements from an existing crack on the bridge from 9 am to 6 pm show that the crack closes as the temperature rises and reopens as it gets colder. Sensors 1 and 5 are partially peeled off from the surface, therefore they are omitted. (**b**) Crack paths on sensors 6–8 are highlighted using a white line. Sensors 2–4 are placed on crack-free surface.

Given the location of crack, the only factor that influences its size in the short term is air temperature. Increase of temperature is supposed to make the crack opening smaller due to thermal expansion of the concrete. The strain readings from 9 am to 6 pm showed that sensors 6–8 sensed significant change in the strain field, as shown in Figure 10a. The negative sign of the strain implies the crack size is reduced compared to the moment when the baseline measurement is taken. A previous study showed that if a crack passes through a thin-film resistive sensor, like in the case of sensors 6 or 8, the crack size increases by 0.04 μm perμϵ in the measured strain [36]. Therefore, it can be estimated that the crack below sensor 6 shrank about 24 μm when the measured strain reached its minimum. As the temperature decreased from its peak value (going back to our baseline measurement temperature), measured strain values started to increase, returning to the baseline values. Although the crack passes through both sensors 8 and 6 in a similar way, sensor 8 is located further away from the point where the pier connects to the embankment, where the maximum stress is expected. Sensor 7 has a lower strain reading since the crack only partially passes through the strain-sensitive area (see Figure 10). The readings from sensors 1 and 5 are omitted because they were partially peeled off from the surface while connecting the sheet to the readout circuit. As expected, the measurements from sensors 2–4 remained constant since they were glued to a crack-free surface (and self-compensated to temperature and thermal strain). They also demonstrate to us again that the sensing sheet needs to cover large areas for reliable detection, and sensors even a few cm away do not sense the damage.

## 5. Conclusions

This paper presented a scalable resistive strain sensing sheet design that was fabricated with the low-cost, high-volume flexible PCB manufacturing technology. The sensing sheet was tested both in the lab and in the field. The sensing sheet’s gauge factor is about 2.1, similar to the reference commercial constantan-based resistive strain gauge. The stability tests showed 3 μϵ drift over 2 days, which is acceptable for the purposes of damage detection. The field tests showed that it is stable and highly sensitive to track the thermally induced mechanical strain change on a pedestrian bridge, with a RMS error measured over 6 sensors of 7 $\mu \epsilon_{rms}$, which is sufficient and suitable for the purposes of damage detection. The tests on the existing crack showed that the sheet can detect the damage when it happens under the strain-sensitive area. While the sensor on the crack recorded −600 μϵ (24 μm decrease in the crack gap), the nearby sensors on crack-free surface did not register significant strain change. This shows that our scalable strain sensing sheet design is a suitable and important component in the damage detection systems.
